# Local-manifold-distance-based regression: an estimation method for quantifying dynamic biological interactions with empirical time series

**DOI:** 10.1098/rsos.231795

**Published:** 2024-07-31

**Authors:** Kazutaka Kawatsu

**Affiliations:** ^1^ Graduate School of Life Sciences, Tohoku University, Sendai 980-8578, Japan

**Keywords:** nonlinear time-series analysis, Jacobian estimation, dynamic stability, ecological forecasting, structural stability

## Abstract

Quantifying species interactions based on empirical observations is crucial for ecological studies. Advancements in nonlinear time-series analyses, particularly S-maps, are promising for high-dimensional and non-equilibrium ecosystems. S-maps sequentially perform a local linear model fitting to the time evolution of neighbouring points on the reconstructed attractor manifold, and the coefficients can approximate the Jacobian elements corresponding to interaction effects. However, despite that the advantages in nonlinear forecasting with noise-contaminated data, these methodologies have a limitation in the Jacobian estimation accuracy owing to non-equidistantly stretched local manifolds in the state space. Herein, we therefore introduced a local manifold distance (LMD) concept, a non-equidistant measure based on the multi-faceted state dependency. By integrating LMD with advanced computation techniques, we presented a robust and efficient analytical method, LMD-based regression (LMDr). To validate its advantages in prediction and Jacobian estimation, we analysed synthetic time series of model ecosystems with different noise levels and applied it to an experimental protozoan predator–prey system with established biological information. The robustness to noise was the highest for LMDr, which also showed a better correspondence to expected predator–prey interactions in the protozoan system. Thus, LMDr can be applied to study complex ecological networks under dynamic conditions.

## 1. Introduction

Various environmental factors drive population and community dynamics. For example, changes in temperature and nutrient influx affect marine and freshwater plankton growth rates [1,2]. Moreover, shifts in species composition modify ecological relationships (e.g. predator–prey, competition and mutualism), affecting species coexistence in natural ecosystems [3–6]. Thus, understanding these biotic and abiotic interactions is crucial for ecological studies. Generally, the following two conventional approaches have been used for this purpose. The first involves manipulative experiments that measure changes in population or community states after altering causal factors [3,7–9]. The second fits the observed data to theoretical or statistical methods, accounting for the effect of the studied interactions on population/community dynamics [10–12]. However, the first approach can be labour-intensive in a natural setting because of the numerous interactions that must be considered [8,9]. Because the high dimensionality of natural ecosystems makes the governing equations indeterminate *a priori* and introduces nonlinearity into the interactions involved [13,14], the straightforward application of the latter approach can also potentially lead to incorrect conclusions about ecological interactions [15,16].

Recent advances in nonlinear time-series analyses have offered promising tools for studying nonlinear phenomena in complex ecosystems. Particularly, the S-map (sequential locally weighted global linear map, [17]) analysis provides a scheme to measure changing biological interactions in non-equilibrium ecological dynamics. This method performs nonlinear forecasting with information about neighbouring relationships among the states on the attractor manifold (a set of state vectors consisting of system variables, such as species density, resource abundance and temperature), which is reconstructed by projecting time-series data of the observed variable(s) onto its (lagged) coordinate space. Specifically, in the S-map procedure, a linear prediction model is sequentially fitted by assigning greater weights to local points near the current state of the attractor [17]. If a model with greater weighting produces a better forecast, the behaviours of the system differ between distant states. Thus, the degree of localization is a good proxy for data nonlinearity [18,19]. Furthermore, as the model coefficients obtained as a by-product of the S-map calculation approximate Jacobian elements (a mathematical representation of interaction strengths, [20]), this method may be used for quantifying the effects of ecological interactions in empirical time series [21–24].

**Figure 1.**
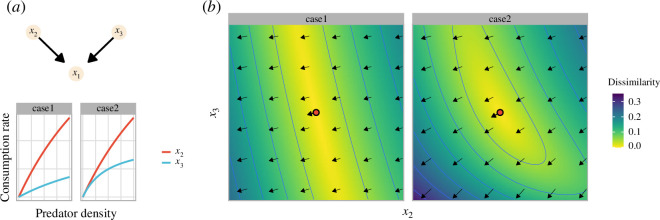
Multi-faceted state dependency of species interaction. (*a*) The top graph is the network of a one-prey–two-predator system. The bottom panel shows the state dependency of the Jacobian coefficients *J*_1*j*_ = ∂*x*_1_/∂*x_j_* (*j* = 2 or 3) on the predator density; ‘case1’ and ‘case2’ at the top of each panel indicates the situation of the more efficient predation of *x*_2_ than *x*_3_ and that of the stronger predator interference within *x*_3_ than *x*_2_, respectively. (*b*) The vector fields based on *J*_1*j*_ at the same prey density *x*_1_ = 0.5 in the *x*_2_ – *x*_3_ space. The arrows correspond to the vectors (*J*_12_ and *J*_13_ at each state) and the colours demonstrate the vector similarity of each state to the red point.

Unfortunately, S-map analysis should still have a limitation in accurately estimating interaction effects owing to the variation in nonlinearity among the interacting factors. This is because different mechanisms can result in the nonlinear nature of biotic and abiotic interactions [25–28]. Therefore, the relative contribution to nonlinearity differs among the distinct processes underlying ecological dynamics (figure 1*a*), and this multi-faceted state dependency necessarily introduces non-equidistantly stretched structures to local manifolds in the coordinate space of observed variables (figure 1*b*). However, the S-map infers the optimum local weighting parameter uniformly, but not individually, for each coordinate (variable) in the state space [17]. Thus, uniform localization may limit the ability of S-maps to estimate the relative contribution to ecological nonlinearity among distinct mechanisms, introducing unignorable systematic biases in estimating Jacobian elements using S-maps (see §2). Although methodological advances have provided several extensions to S-maps [21,29–31], the constraint of uniform localization remains.

To overcome this limitation, we introduced the concept of local manifold distance (LMD), which enables calculating a localization weighting individually for each variable. Therefore, applying an optimized LMD to ecological time series allows the multi-faceted nonlinearity of the different factors underlying the system to be accounted for, thus, improving the estimation accuracy of ecological interaction strengths. In this study, by combining LMD with a regularization technique and a parallel computing algorithm, we developed a noise-robust, computationally efficient analysis of locally weighted regression (hereafter, an LMD-based regression, LMDr). To demonstrate the performance of the LMDr in prediction and Jacobian estimation problems, we first analysed synthetic time series with several ecological models under different process noises and observation errors. We then applied the method to a protozoan predator–prey system, in which the biological information of the interaction was well-established. The findings indicated that the LMDr is a promising tool not only in investigating species interactions in natural ecosystems, but also in estimating other dynamical properties, such as a stability of fluctuating ecological networks.

## 2. Methods

### 2.1. Nonlinear nature of ecological interaction

Ecological interaction strength is commonly defined as the change in the population growth rate of a species under a slight change in the state of interacting biotic and abiotic factors [20]. This definition is mathematically equivalent to the Jacobian element, that is the partial derivative of a species’ growth rate with respect to the system variable, and it is often evaluated at equilibrium in conventional ecological modelling. However, natural ecosystems are generally in non-equilibrium states, and the interaction effects fluctuate temporally with shifts in causal biotic/abiotic variables (i.e. state dependency of the Jacobian) [21]. Additionally, various characteristics of community dynamics, including rapid intrinsic growth rates, strong interaction effects, density dependence, functional response and network complexity, cause nonlinear dynamics [25–28]. Thus, ecological interaction effects exhibit variable responses to different system’s states.

To illustrate this, consider a simple one-prey–two-predator system with predator-dependent functional responses [32,33], wherein the density of prey species 1, predator species 2 and predator species 3 are denoted by *x*_1_, *x*_2_ and *x*_3_, respectively. As examples, we particularly consider the two cases: (i) the more efficient predation of *x*_2_ than *x*_3_ with the same predator interference; and (ii) the stronger predator interference within *x*_3_ than *x*_2_ with the same predation efficiency (figure 1*a*). Thanks to the known model structure (see the electronic supplementary material), the theoretical values of the interaction strengths/Jacobian coefficients are calculated, and their nonlinear behaviour with respect to species density is easily visualized using the vector field. As shown in figure 1*b*, the predation effects on prey species, ∂*x*_1_/∂*x*_2_ and ∂*x*_1_/∂*x*_3_, are not constant but vary depending on the combination of predator species density. Notably, owing to the stronger predation efficiency of *x*_2_ than *x*_3_ for case 1 and the weaker interference in *x*_2_ than *x*_3_ for case 2, the changes are steeper along the *x*_2_-axis than the *x*_3_-axis for both cases (figure 1*b*). Thus, the Jacobians (∂*x*_1_/∂*x*_2_, ∂*x*_1_/∂*x*_3_) are more sensitive to the density changes in *x*_2_ than those in *x*_3_, indicating that local (differentiable) manifolds where linear approximation of the system is applicable exist in a stretched/contracted form in the coordinate space composed of the observed variables (or their lags). These examples highlight that various mechanisms induce the multi-faceted state dependency into the system and the need to consider the non-equidistant neighbourhood relationships in the vector field when building a prediction model for population/community dynamics.

### 2.2. Concept of local manifold distance-based regression

**Figure 2.**
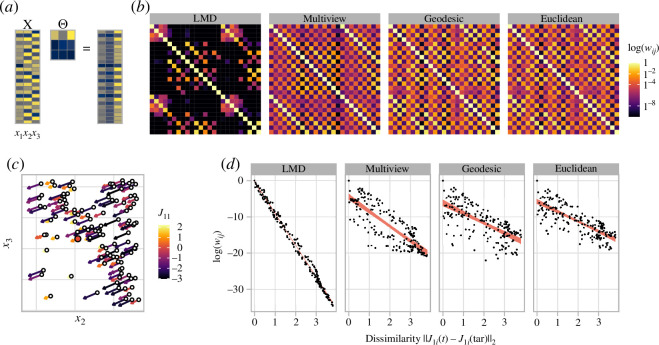
Schematic of the concept of LMDr analysis. (*a*) The effect of locality matrix **Θ** on the data magnification. The yellower colours indicate the higher values in the element of the raw embedding matrix *x*, the locality matrix **Θ** and the weighted embedding matrix. (*b*) The difference in the weighting matrix *w*(**Θ**) and those with different distance measure. The distance measures used in the weighting matrices are denoted at the top of each panel (LMD, multi-view, geodesic and Euclidean distance). The colour of each grid corresponds to the logarithm log(*w_ij_*) in the weighting matrix. (*c*) The left panel is the *J*_1*j*_ calculated from the one-prey–two-predator model of case 1. The arrows correspond to the magnitude of (*j*_12_, *j*_13_) and the colours show that of *J*_11_. The right panels show the relationship between the vector dissimilarity (*x*-axis) and the logarithmic weights (*y*-axis) of each state from the target depicted in the left panel (white circle and red point, respectively). The line ranges are the 95% confidence interval in the linear regression.


For this end, we here introduced the locality matrix, **Θ** = {*θ_ij_*}∈ ℝ*^E^* ^× *E*^ (*E* indicates the embedding dimension or the number of state variables), into distance calculation so that the distance measure can stretch/contract in any direction in the state space. Specifically, the **Θ** independently controls the weights of direction in the embedding matrix **X**∈ ℝ*^m^* ^× *E*^ (figure 2*a*), where *m* is the available data size, yielding the locally weighted distance **D**(**Θ**) = {*d_ij_* (**Θ**)}∈ ℝ*^m^* ^× *m*^ with:

(2.1) $$\begin{array}{c} d_{ij}\left(\Theta \right)={\left\|\left(x_{i}-x_{j}\right)\Theta \right\|}_{2}\text{.} \end{array}$$

Here, **x** *_i_* and **x** *_j_* indicates the *i*th and *j*th row in the matrix **X**, respectively, and ||·||_2_ denotes the L2 norm of the vector. Subsequently, the importance of each data point in fitting the local linear model is determined by the weighting matrix **W**(**Θ**) = {*w_ij_* (**Θ**)} ∈ ℝ*^m^* ^× *m*^ as:


(2.2) $$\begin{array}{c} w_{ij}\left(\Theta \right)=\exp\left(-\frac{d_{ij}\left(\Theta \right)}{{\overset{-}{d}}_{i}\left(I\right)}\right)\text{,} \end{array}$$

where **I** is an *m* × *m* identity matrix, *d_ij_* (**I**) corresponds to the Euclidean distance between states *i* and *j*, and *d̅_i_* is the mean distance of the *i*th row in the distance matrix.

Similarly, the S-map is a nonlinear forecasting method with a locally weighted linear regression, and its methodological advances are currently ongoing. In particular, the two recent extensions use a distance measure other than the Euclidean distance in calculating local weights to neighbour points. One is the multi-view distance regularized (MDR) S-map, in which the neighbourhood relationship is calculated by averaging numerous distances measured in possible low-dimensional attractors composed of observed variables, allowing the reconstruction of high-dimensional ecological networks [30]. The other S-map variant uses geodesic distance, which measures the shortest path between two states on an attractor with accounting for the low-dimensional geometry of the attractor (hereafter, GD S-map) [31]. However, in these S-map variants, the weighting matrix is calculated with the distance matrix for each method and a locality parameter that is a scalar but not a matrix as:


(2.3) $$\begin{array}{c} w_{ij}\left(\theta \right)=\exp\left(-\theta \frac{d_{ij}}{{\overset{-}{d}}_{i}}\right)\text{,} \end{array}$$

where *d*_*ij*_ is the distance between states *i* and *j* for each S-map variant (i.e. Euclidean distance for the S-map, multi-view distance for the MDR S-map and geodesic distance for the GD S-map). Thus, this uniform localization of the S-map variants yields the weighting matrices significantly different from that of the LMDr (figure 2*b*). As a result, in an example of the one-prey–two-predator system, the weighting to neighbour points strongly correlated with the Jacobian similarity between the states in the LMDr, but the relationship was weaker for the S-map variants (figure 2*c*).

### 2.3. Implementation of local manifold distance-based regression

Based on the weighting matrix **W**(**Θ**) obtained by equation (2.2), the LMDr sequentially calculates the linear prediction model:


(2.4) $$\begin{array}{c} {\hat{x}}_{i}\left(t_{k}+1\right)=c_{i0}+\sum_{j=1}^{E}c_{ij}x_{j}\left(t_{k}\right)\text{,} \end{array}$$

where *t_k_* is the *k*th time to be predicted and *x_i_* indicates the *i*th element of the *E*-length state vector **x** *_k_*. Specifically, the *E*-length coefficient vector **c** *_k_* is obtained by solving the linear equation:


(2.5) b=Ac,

where **b** = {*w_kj_* (**Θ**)·*x_i_* (*t_j_* + 1)} ∈ ℝ*^m^* and **A** = {*w_kj_* (**Θ**)·**x** *_j_*} ∈ ℝ*^m^* ^× *E*^. In the original S-map algorithm [17,21], this equation is solved with the singular value decomposition (SVD). However, the SVD approach is vulnerable to data with strong process noise, resulting in overfitting in the Jacobian estimation [29]. A recently developed S-map variant, known as the regularized S-map, addresses this issue by introducing a regularization term penalizing larger coefficients of the local linear model [29]. There are several types of regularizations; in this study, we used the ridge regression (also known as Tikhonov regularization) because of its convenience in implementing the locality matrix **Θ**. In particular, equation (2.5) is replaced by the following minimization problem:


(2.6) $$\begin{array}{c} {\hat{c}}_{k}=\underset{c\in R^{E}}{argmin}\left(\frac{1}{n}{\left(x^{\prime }-Xc\right)}^{T}W_{k}\left(x^{\prime }-Xc\right)+\lambda {\left\|c\right\|}_{2}\right)\text{,} \end{array}$$

where **x**′ ={*x_i_* (*t* + 1)} and **W** *_k_* = diag(*w_k_* (**Θ**)). The parameter *λ* controls the magnitude of penalization and improves the instability of matrix inversion, enabling an analytical solution of equation (2.6) as **ĉ** *_k_* = (**X** ^T^ **W** *_k_* **X** + *nλ* **I**)^–1^ **X** ^T^ **W** *_k_* **x**′.

Contrastingly, determining the optimal locality matrix **Θ** cannot be solved analytically. Therefore, approximating the optimal **Θ** of using a numerical approach is necessary. Many numerical methods have been proposed for global optimization of complicated problems such as the gradient-descent-based optimization methods [34]. However, these methods risk falling into local optima and may not be suitable for simultaneous optimization of multiple variables in **Θ**. Instead, we adopted a temperature-parallel simulated annealing (TPSA), which is an extension of the simulated annealing (SA) algorithm for parallel computing. SA is a probabilistic technique for solving combinatorial optimization problems that emulate the controlled cooling of a heated material to alter its physical properties [35]. Specifically, in the SA process, various states are randomly investigated at high temperatures and selected to minimize the cost function as the temperature decreases. With an appropriate annealing schedule, an SA run is statistically guaranteed to reach the global optimum [35,36]. The TPSA, instead of a temporal cooling schedule, probabilistically exchanges information collected from multi-process annealing at fixed but different temperatures, thereby efficiently improving the overall rate of convergence to the solution [37,38].

The specific procedures of the LMDr consists of several steps. First, a TPSA execution environment was set up as *k* SAs with different temperatures ranging from [*T*_min_, *T*_max_]. The initial values are set to *λ*_old_ = 0 and **Θ**_old_ = **I** for each SA run, and the prediction performance was evaluated with a root mean squared error of the current state, RMSE_cur_. Then, the mutation process was done as *λ*_new_ = |*λ*_old_ + *δ|* and **Θ**_new_ = |**Θ**_old_ + **Δ**| (**Δ** = {*δ*} and *δ*~*N*(0, *σ_δ_*)), and the prediction performance RMSE_new_ was also calculated. The mutation parameters were always selected as the new ones when Δ*E* = RMSE_new_ – RMSE_old_ < 0 and otherwise with the probability as:


$$\begin{array}{c} P\left(T_{i}\right)=\exp\left(-\frac{\Delta E}{T_{i}}\right)\text{,} \end{array}$$

where *T_i_* indicates the temperature of *i*th SA. This process was iterated for 100 times for each SA run, and the parameter exchange between SAs with adjacent temperatures was performed with the probability as:


$$\begin{array}{c} P\left(T_{i},T_{j}\right)=\left\{ \begin{array}{l} \begin{array}{cc} \;\;\;\;\;\;\;\;\;\;\;\;\;\;\;\;\;\;\;\;\;\;\;\;\;\;\;\;\;1 & if\;\Delta T\cdot \Delta E<0\\ \exp\left(-\frac{\Delta T\cdot \Delta E}{T_{i}\cdot T_{j}}\right) & otherwise \end{array} \end{array}\right.\text{,} \end{array}$$

where Δ*T* = *T_i_ – T_j_*. The exchange process was also iterated for 100 times. We implemented the LMDr with this procedure in an R package ‘andsr’ (electronic supplementary material) and applied it to the following analyses.

### 2.4. Performance of local manifold distance-based regression in Jacobian estimation

We first evaluated the performance of the LMDr in Jacobian estimation by analysing synthetic time-series data of five nonlinear dynamical systems, where the model structure is known *a priori*. The models were selected from the literature of dynamical systems and ecological studies, which show chaotic dynamics with different model complexities (3–20 variables), two being continuous and three being discrete systems (for the detail of each system, see the electronic supplementary material). We generated the time-series data to contain a certain magnitude of process noise and observation error based on the following procedure.

A continuous-time dynamical system (the Lorenz63 and 5-species food-chain model) with stochastic process noise is in general described as the following ordinary differential equation:


(2.7) $$\begin{array}{c} \frac{dx}{dt}=f\left(x+\delta \right)\text{,} \end{array}$$

where **f** is a set of nonlinear equations for each system and **x** is an *n*-length vector of each state variable. The noise component is expressed by an *n*-length vector **δ**, where the *i*th component *δ_i_* is randomly assigned from a normal distribution with a mean 0 and standard deviation *σ*_proc_ × SD(*x_i_*), where SD(*x_i_*) is the standard deviation of the variable *i*. Time-series data used for the analysis were generated in the following two steps: (i) integrating equation (2.7) with the fourth-order Runge–Kutta approximation with the integration step 0.01; and (ii) sampling the data at an appropriate frequency to enable the reconstruction of the dense attractor. For a discrete-time dynamical system (the one-prey–two-predator system with predator dependent functional responses, 5-species coupled logistic map and 20-species Ricker model) with stochastic process noise is also described as the form of difference equation as:


(2.8) $$\begin{array}{c} x\left(t+1\right)=f\left(x\left(t\right)+\delta \left(t\right)\right)\text{,} \end{array}$$

where **x**(*t*) and **δ**(*t*) is the discretized version of state-variable and process noise vector described above, respectively. The time-series data used for the analysis were generated with equation (2.8). For both continuous- and discrete-time systems, the generated time-series data were further contaminated with the observation error **ϵ**(*t*) = {*ϵ_i_* (*t*)}, which is randomly assigned from a normal distribution with a mean 0 and standard deviation *σ*_obs_ × SD(*x_i_*).

For the analysis, applying the LMDr to the generated dataset, we performed nonlinear prediction and Jacobian estimation. Specifically, we first built a data matrix **X** ∈ ℝ*^m^* ^× *n*^ where each row comprised the state vector **x** *_t_* = {*x_i_* (*t*)} (*m* and *n* stand for the data length and the system size, respectively). Then, we optimized the locality matrix **Θ** that gives the maximum prediction skill (evaluated by the RMSE) for each state variable with the TPSA algorithm and leave-one-out cross-validation. Although the elements of **Θ** can be any positive real number, this assumption yields a number of (*n* × *n*) free parameters. Thus, we set the off-diagonal elements *θ_ij_* = 0 in the analysis if not otherwise specified. Based on the best LMDr model, we performed nonlinear forecasting and Jacobian estimation.

The procedure of Jacobian estimation slightly differed between continuous- and discrete-time systems. Specifically, for the continuous-time system, we calculated the Jacobian matrix by taking partial derivatives with equation (2.7) as:


(2.9) $$\begin{array}{c} J\left(t\right)=\frac{\partial f}{\partial x}\left(t\right)\text{.} \end{array}$$

Then, equation (2.9) can be discretized by taking exponential matrix with integration time 0.01 and sampling frequency *τ* as:


(2.10) $$\begin{array}{c} K\left(t\right)=e^{0.01\tau J\left(t\right)}=\sum_{i=0}^{\infty }{\left(0.01\tau J\left(t\right)\right)}^{i}\text{.} \end{array}$$

For the discrete-time system, the Jacobian matrix was obtained with equations (2.8) and (2.9). The true Jacobian elements were based on these equations with the time-series data without error and the performance of Jacobian estimation was evaluated by calculating the Pearson’s correlation coefficient *ρ* between the true Jacobian elements and the corresponding model coefficients of the best LMDr model.

To compare the performance of nonlinear forecasting and Jacobian estimation, we also applied the above analysis to the S-maps with different distance measures (i.e. Euclidean, multi-view and geodesic distance). In order to compare only the difference in distance measures, regularization was not considered in this analysis (for more details, see the electronic supplementary material). Prior to the analysis, we prepared the data size *m* = 200 and 300 time series for the discrete- and continuous-time systems and performed the above procedure. The simulation was conducted under different magnitudes of process noise/observation error (σ_proc_, σ_obs_∈ (0.001, 0.051, 0.101)) and iterated 10 times for each noise setting.

### 2.5. Analysis of experimental time series

We then tested the prediction skill and Jacobian estimation performance of the LMDr in a real ecological time series. For the analysis, we used a dataset of the protozoan system of *Paramecium* and *Didinium* [39,40]. In this system, the *Paramecium* populations were grown in Cerophyl medium, a resource of bacterial populations on which the *Paramecium* feed [40], with a concentration of CC0.375 ≈ 0.68 g l^-1^ or CC0.5 ≈ 0.90 g l^-1^. The *Didinium* populations feed on their prey, *Paramecium*, in the culture [39,41,42].

The exact equation for this system was also unknown like other natural ecosystems. However, thanks to established biological information, we could qualitatively hypothesize the sign (i.e. negative/positive) and magnitude of the *Paramecium*–*Didinium* interaction, and its form of the functional response. Specifically, since this system can be regarded as a predator–prey system with *Didinium* as the predator and *Paramecium* as the prey, the effect of the *Didinium* population on the *Paramecium* population is expected to be negative and the opposite effect is to be positive. In addition, as the *Didinium* and *Paramecium* individuals have similar body sizes and exhibit relatively random movements [39,42], the per capita ability of the predator to consume prey individuals is limited, leading to a functional response that predation efficiency saturates with increasing prey density. Furthermore, Veilleux [39] intensively investigated how the nutritional condition affects the *Paramecium–Didinium* interaction with laboratory experiments and found that: (i) the low Cerophyl concentration (CC0.375) reduces the density and nutritional quality of the *Paramecium* population as the resource for the *Didinium* population; and (ii) this starvation in the prey results in reduced per capita attack rate in the predator.

In summary, we can hypothesize the *Paramecium–Didinium* interaction as follows: (i) the negative (positive) population-level effect of *Didinium* (*Paramecium*) on *Paramecium* (*Didinium*); (ii) the predation efficiency with a functional response saturating with an increase in the prey density; (iii) the weaker positive (negative) effect from the prey (predator) in the CC0.375 than CC0.500; and (iv) the weaker functional response in the CC0.375 than CC0.500. To test these hypotheses, we applied the LMDr and the MDR S-map to the three experimental datasets of the *Paramecium–Didinium* system conducted under two Cerophyl conditions (CC0.500 for experiment 1 and 3; CC0.375 for experiment 2). For each experimental setting, the density of *Paramecium* and *Didinium* populations was monitored every 12 h for 35 days in experiment 1, 32 days in experiment 2 and 25.5 days in experiment 3. The previous studies reported that there were some sampling errors in counting the individual number, despite the relatively constant experimental setting [39,40]. Thus, reflecting this sampling errors would increase the robustness of the results, and we performed the analysis as the following procedure.

First, the time-series data of *Paramecium* (Pa) and *Didinium* (Dn) were standardized to zero mean and unit-variance by subtracting the mean and dividing by the standard deviation for each species. Then, the optimal embedding dimension *E* of the prey and predator time series was selected from 2 to 10 for each experiment, which produces the best forecast skill (evaluated by RMSE) with simplex projection, a standard nonlinear forecasting method [43]. Based on the optimal *E*, we reconstructed a multi-variate embedding matrix **X**, where the first two columns comprised Pa(*t*) and Dn(*t*), and if *E* > 2, the time lags of the prediction target (i.e. Pa(*t* – 1), …, Pa(*t* – (E – 2)) or Dn(*t* – 1), …, Dn(*t* – (*E* – 2))) were added. Applying the LMDr and the MDR S-map to the ‘clean’ (i.e. with no sampling errors) time-series data, we obtained the prediction skills (RMSE), estimated Jacobian coefficients and those relationships with the species density, which were used to the baseline for the analysis. Finally, the noise contaminated time series were generated by adding sampling errors with the standard deviations σ_Pa_(*t*) = 0.417 × Pa(*t*)^0.5^ and σ_Dn_(*t*) = 0.164 × Dn(*t*)^0.5^ for the *Paramecium* and *Didinium* data, respectively. The noise-contaminated data were also analysed according to the above procedure, where they were repeated 100 times.

The LMDr was performed with a function ‘find_best_theta’ in the R package ‘andsr’, which implements the procedure described above. For the MDR S-map, the multi-view distance was calculated based on the procedure described in the electronic supplementary material. Then, based on the obtained multi-view distance, the local linear regression was done with equations (2.3) and (2.6) (i.e. the ridge regression with the uniform localization). The optimal value of θ was searched with the grid-search algorithm from 0 to 20 by 0.1, and the ridge regression was done with the function ‘cv.glmnet’ in an R package ‘glmnet’ v. 4.1–8. All the analyses were performed using R v. 4.3.1 (R Development Core Team 2023).

## 3. Results

**Figure 3.**
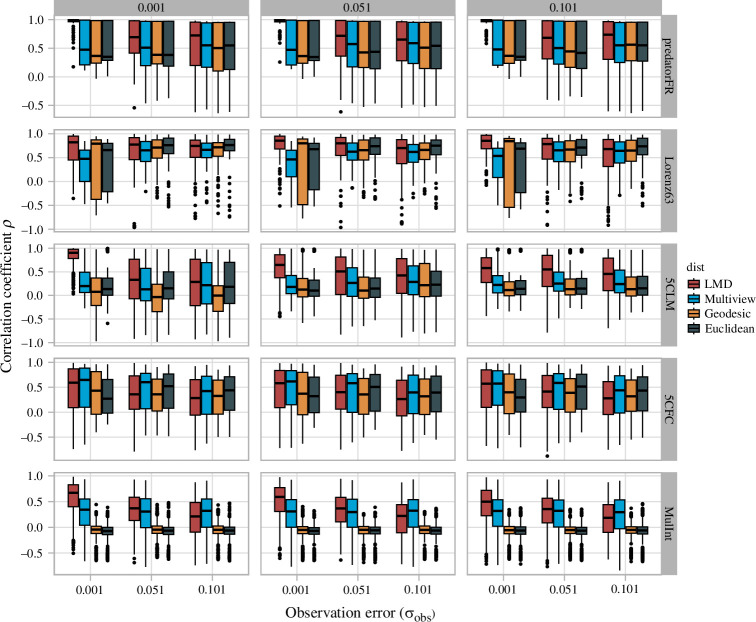
The effect of observation error and process noises on the Jacobian estimation skill of nonlinear forecasting methods with different distance measures (LMD, multi-view, geodesic and Euclidean distance). The estimation skill was calculated with the Pearson’s correlation coefficient between target Jacobian coefficients *J_ij_* and its approximation *Ĵ_ij_* for each method. The solid lines within the boxplots represent the median of the estimation skill; the lower and upper hinges in the boxplots correspond to the first and third quantiles; the upper and lower whisker extends from the hinge to the largest and smallest value no further than 1.5 × inter-quantile range (the distance between the first and third quantiles). The results of the different process noises (*σ*_proc_ = 0.001, 0.051 and 0.101) are aligned column-wisely, and those of the different models are aligned row-wisely. The ‘predatorFR’, ‘Lorenz63’, ‘5CLM’, ‘5CFC’ and ‘MulInt’ correspond to the one-prey–two-predator with predator-dependent functional responses, Lorenz63 system, the five species coupled logistic map, the five species food-chain model and the 20 species Ricker model, respectively.

**Figure 4.**
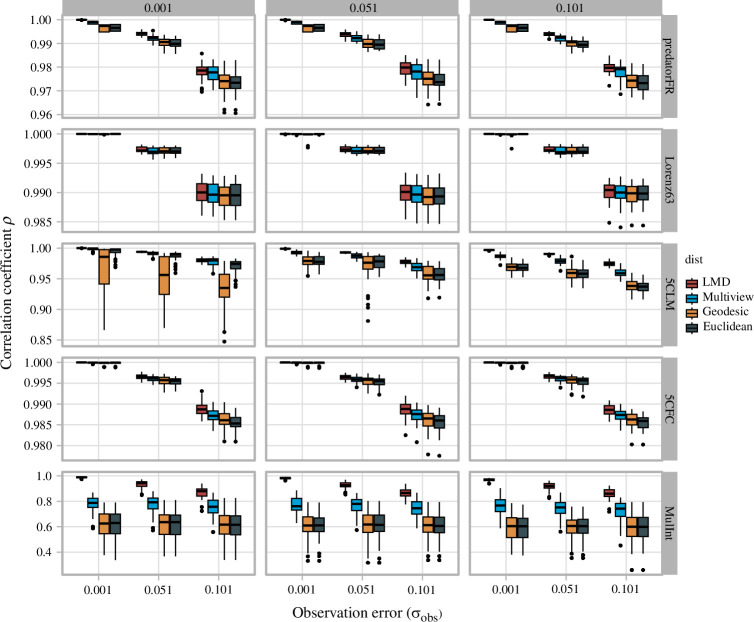
The effect of observation error and process noises on the prediction skill of nonlinear forecasting methods with different distance measures (LMD, multi-view, geodesic and Euclidean distance). The prediction skill was calculated with the correlation coefficient *ρ* between target variable *x_i_* and its prediction *x̂_i_*. The lines in the boxplots, the column and row order of each panel are consistent with figure 3.

Figure 3 shows the performance of LMDr in the Jacobian estimation compared to the S-maps with different distance measures (multi-view, geodesic and Euclidean distance). The performance degraded as observation errors and process noises increase. In particular, the robustness to noise was the highest for the LMDr, followed by the S-map with multi-view distance, that with geodesic distance and Euclidean distance in that order. The result also showed that the estimation skills of these methods were more vulnerable to the increased observation error in the continuous-time systems (Lorenz63 and the 5-species coupled food-chain model), weakening the above-mentioned tendency (figure 3). Additionally, we investigated the performance of nonlinear forecasting. As shown in figure 4, the performance also deteriorated with increasing observation errors and process noise, and the predictions tended to be best with the LMDr and worse with the multi-view, geodesic and Euclidean distance in that order. Similar to the Jacobian estimation, this trend was a little less detectable in the continuous-time systems (figure 4).

**Figure 5.**
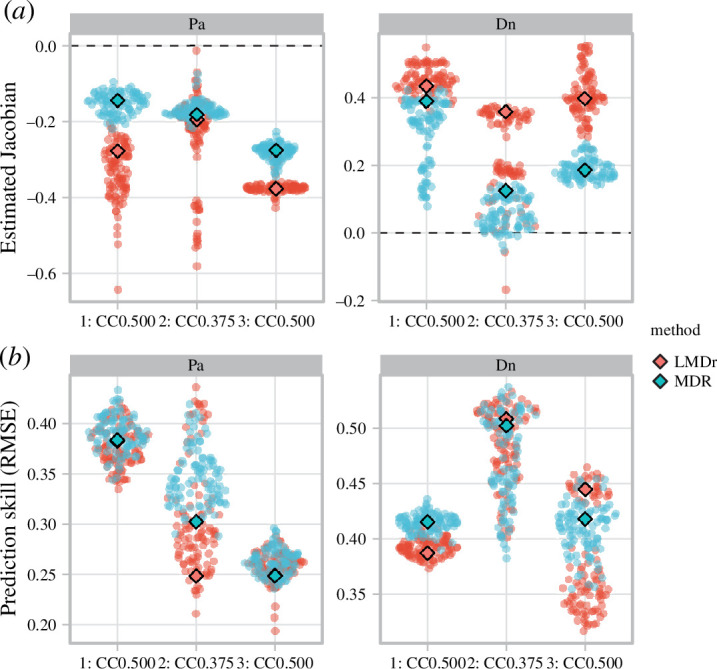
Estimated Jacobian (*a*) and prediction skill (*b*) of the *Paramecium–Didinium* dataset for LMDr and MDR S-map (denoted by red and blue colours, respectively). The prediction target and the interaction recipient species (Pa, *Paramecium*; Dn, *Didinium*) are denoted at the top of each panel. The horizontal axis indicates the experimental identity of the data analysed. The diamonds represent the results of the original time series, and the swarm-plots demonstrate the distribution of the results of noise-added time series. Estimated Jacobian corresponds to the effect of the other protozoan on the prediction target, and the dashed lines highlight the zero effect of species interaction.

Figure 5 summarizes the LMDr and the MDR S-map analyses of the time-series data from the *Paramecium–Didinium* experiment. For the sign of estimated Jacobian coefficients, both methods yielded similar results for each experiment; Jacobian coefficients ∂Pa(*t* + 1)/∂Dn(*t*) corresponding to the effect of *Didinium* on *Paramecium* were negative (left panel in figure 5*a*) and those corresponding to the effect of *Paramecium* on *Didinium* (∂Dn(*t* + 1)/∂Pa(*t*)) were positive (right panel in figure 5*a*). However, the relationship between the magnitude of Jacobian coefficients and the nutritional conditions differed slightly between the two methods. For the estimated coefficients ∂Pa(*t* + 1)/∂Dn(*t*), its magnitudes were more negative in the CC0.5 setting (experiments 1, 3) than in the CC0.375 setting (experiment 2) in the LMDr results, while those were more negative in the order of experiments 1, 2 and 3 in the MDR S-map results (left panel in figure 5*a*). The Jacobian coefficients ∂Dn(*t* + 1)/∂Pa(*t*) were less positive in the CC0.375 setting than the CC0.5 setting for both methods; however, those in the CC0.375 were close to zero value for the MDR S-map results (right panel in figure 5*a*). For the nonlinear prediction, both methods showed similar prediction skills for the *Paramecium* and *Didinium* time series (figure 5*b*).

**Figure 6.**
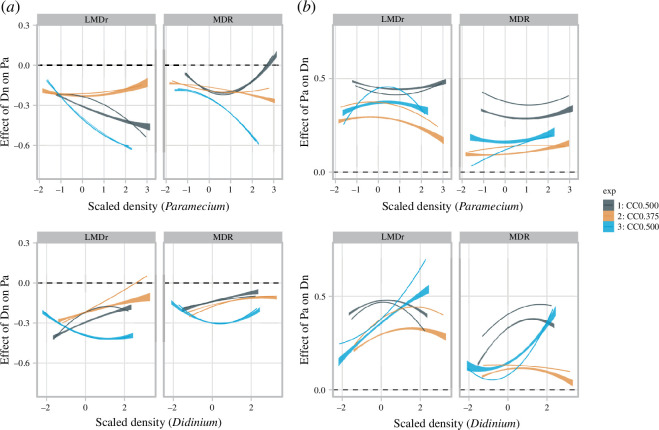
Functional relationship between the protozoan species densities (horizontal axis) and estimated effects of *Didinium* on *Paramecium* (*a*) and those of *Paramecium* on *Didinium* (*b*). The different colours correspond to the experimental identity (red, experiment 1 with CC0.5; green, experiment 2 with CC0.375; blue, experiment 3 with CC0.5). The dashed lines highlight the zero effect of species interaction. The bold lines represent the quadratic polynomial regression to the results of the original time series, and the shaded regions are the 95% confidence interval of the quadratic polynomial regression to the results of noise-added time series.

The difference in the Jacobian estimation between the two methods was also prominent in the functional relationships between the estimated coefficients and species densities. Specifically, the negative effects of *Didinium* on the *Paramecium* population were saturated with an increase in the number of *Paramecium* individuals in the CC0.5 setting for the LMDr, whereas the relationship was not consistent between experimental settings for the MDR S-maps (figure 6*a*, top panels); they tended to attenuate with increasing *Didinium* density for both methods (figure 6*a*, bottom panels). The positive effects of *Paramecium* on *Didinium* were less dependent on the increasing number of *Paramecium* individuals for both methods (figure 6*b*, top panels). The effects of *Paramecium* on the *Didinium* population showed the convex curves for the LMDr, indicating the saturation with increasing *Didinium* density, while those even accelerated with the *Didinium* density in the experiment 3 for the MDR S-map (figure 6*b*, bottom panels).

## 4. Discussion

Ecological dynamics are driven by various environmental factors with different nonlinearities (figure 1), making it challenging to measure the effects of these interactions in natural settings. Recent advances in nonlinear time-series analyses, including S-map variants, have provided promising tools to address this issue [21,29–31]. However, even the S-map variants have a limitation in estimating the interaction effects of Jacobian elements because they use uniform localization, that is they optimize a local weighting parameter uniformly but not individually for each variable. To address this limitation, we developed a nonlinear time-series analysis, the LMDr, which extends the calculation of local weighting in any direction of the state space to reproduce the flexible geometry of the vector field (figures 1 and 2). By applying the LMDr to the time-series data of model ecosystems and a real protozoan experiment, we demonstrated its advantage in investigating nonlinear forecasting and estimating interaction effects in a noisy ecological time series, as described below.

The analysis of synthetic data with observation errors and process noise showed that the LMDr outperformed the S-map variants with uniform localization in nonlinear forecasting and quantification of ecological interactions (figures 3 and 4). The additional analyses implied that the benefits of the LMDr exist even for a relatively small dataset, and it also requires less computational cost than the other S-map variants, such as MDR S-map, for large datasets (see the electronic supplementary material). Note that these results depended on the dynamical characteristics of the model ecosystems. Specifically, the advantages of the LMDr in nonlinear forecasting and Jacobian estimation are less detectable for the continuous-time systems. This would be because these systems are vulnerable to the observation errors (figure 3). By contrast, for the discrete-time systems, the advantage of the LMDr in nonlinear forecasting and Jacobian estimation tended to be more pronounced with an increase in the model complexity (figures 3 and 4). In summary, these findings indicated that the LMDr can be used to study ecological interactions in natural settings that essentially involve process noise and observation errors.

The analysis of the *Paramecium*–*Didinium* dataset demonstrated the further advantage of LMDr not only in nonlinear forecasting (figure 5*b*) but also in estimating interaction effects (figures 5*a* and 6) in real ecological time series. Both the LMDr and the MDR S-maps reproduced the signs of interaction effects corresponding to the expectation of a predator–prey relationship (i.e. the negative predator effects of *Didinium* on *Paramecium* and the positive feeding effects of *Paramecium* on *Didinium*). However, the LMDr seemed to be more in line with the expected relationships between the nutrient conditions and the predator–prey interactions: the mitigation of the predation effect on the *Paramecium* population and the decrease in the consumption rewards to the *Didinium* population in the nutrient-poor conditions (figure 5*a*). In fact, the previous study [39] reported that the *Paramecium* individuals reared in the poor nutritional condition also showed low quality as a resource of the *Didinium* individuals, reducing the per capita attack rate. Additionally, the LMDr results were more consistent with the expected functional response. In the *Paramecium*–*Didinium* system, as predator and prey individuals have comparable body sizes and exhibit relatively random movements [39,42], feeding behaviours is well-approximated by a Holling type II functional response. The type II functional response is a predation effect saturated by prey density, which is a better described by the LMDr results than the MDR S-maps (top panels in figure 6*a*). A previous study [40] suggested that predator interference owing to local prey depletion reduces feeding benefits under higher predator densities, which was also pronounced in the LMDr results (bottom panels in figure 6*b*).

An accurate Jacobian estimation of the LMDr may shed light on important issues in ecological studies. For example, changes in the sign/magnitude of interaction effects are expected in various relationships, such as competition and mutualism, as well as in predator–prey relationships, implying the prevalence of context- and density-dependent interactions in nature [44,45]. Recent theoretical studies suggest that the density dependence where the interaction effect weakens with the density of interaction recipient stabilizes the community dynamics in harmful and beneficial interaction networks [46–48]. Thus, applying the LMDr to the time series of natural multi-species community data may elucidate how density dependence plays a role in the persistence of complex ecosystems. Additionally, Jacobian elements estimated with empirical time series can be used to analyse a ‘stability’ of the dynamical system [22,49–51]. However, the estimation error of Jacobian elements would yield unignorable biases in calculating such stability measures [52–54]. The application of the LMDr mitigates this limitation, expanding the possibility of stability analysis in ecological studies.

Future studies on nonlinear forecasting and Jacobian estimation can use the relevance of the LMDr to the recently developed MDR S-map [30]. Natural ecological networks would consist of a number of interacting species and physical environments. For such high-dimensional systems, the number of observed variables or optimal embedding dimension might be smaller than the entire system, yielding significant errors in inferring interaction strengths [30]. As we used the entire variables to construct the attractor in the analysis of synthetic time series, the LMDr might suffer this dimensionality issue. By contrast, a multi-view distance of the MDR S-map algorithm, which is an ensemble of distance matrices measured in various attractors reconstructed with a possible subset of causal variables, is calculated to determine neighbouring relationships among high-dimensional data points. Because the attractors are selected based on their forecasting skills, the MDR S-map enables the reconstruction of high-dimensional interaction networks and improves S-map prediction [30]. In our analysis, the MDR S-map exhibited the second-best performance in terms of prediction and Jacobian estimation (figures 3 and 4). Importantly, the calculation of the multi-view distance can be integrated into the LMDr algorithm, indicating that there is room for improvement in forecasting/Jacobian estimation in high-dimensional ecological data. Specifically, it can be implemented using the following steps: (i) optimizing the LMDr prediction for each possible attractor, (ii) calculating the average LMD matrices, and (iii) performing a nonlinear prediction with the ensemble LMD. However, this procedure would be computationally time-consuming and requires an effective implementation with high-performance computation, such as Graphical Processing Unit parallelization [54].

In this study, we developed the LMDr and applied it to the time-series dataset of model and real ecosystems. The results demonstrated the advantage of the method for nonlinear forecasting/Jacobian estimation and are reliable for application to various ecological issues. The LMDr can be used to analyse time series obtained from various ecosystems and by different monitoring methods, such as remote sensing [52,53] and eDNA sampling [55]. Thus, applying the LMDr for accumulating ecological time series will advance the understanding of complex interaction networks and ecological forecasting.

## Data Availability

Data and relevant code for this research work are stored in GitHub: [56] and have been archived within the Zenodo repository: [57]. Data is also available in the electronic supplementary material [58].
